# Regulatory T Cell and Forkhead Box Protein 3 as Modulators of Immune Homeostasis

**DOI:** 10.3389/fimmu.2017.00605

**Published:** 2017-05-26

**Authors:** Leonn Mendes Soares Pereira, Samara Tatielle Monteiro Gomes, Ricardo Ishak, Antonio Carlos Rosário Vallinoto

**Affiliations:** ^1^Laboratório de Virologia, Instituto de Ciências Biológicas, Universidade Federal do Pará, Belém, Pará, Brazil; ^2^Programa de Pós-Graduação em Biologia de Agentes Infecciosos e Parasitários, Instituto de Ciências Biológicas, Universidade Federal do Pará, Belém, Pará, Brazil

**Keywords:** forkhead box protein 3, regulatory T cell lymphocytes, regulatory status, immunotolerance, immune homeostasis

## Abstract

The transcription factor forkhead box protein 3 (FOXP3) is an essential molecular marker of regulatory T cell (Treg) development in different microenvironments. Tregs are cells specialized in the suppression of inadequate immune responses and the maintenance of homeostatic tolerance. Studies have addressed and elucidated the role played by FOXP3 and Treg in countless autoimmune and infectious diseases as well as in more specific cases, such as cancer. Within this context, the present article reviews aspects of the immunoregulatory profile of FOXP3 and Treg in the management of immune homeostasis, including issues relating to pathology as well as immune tolerance.

## Introduction

The establishment of an immune response occurs due to fortuitous stimuli generated by an unlimited set of extrinsic and intrinsic factors of the organism. In order to be launched, a highly interactive network must be instituted among the innate components of immunity, which will recognize molecular patterns associated with the pathological condition. The design of the response follows a complex recruitment of specialized cells (1–3) with the components of adaptive immunity, which specifically regulate effector cell response through the production of pleiotropic immunologic mediators (4, 5).

The success of the immune response is accompanied by the potential risk in developing damage and autoimmune reactions against the organism. The loss of the discrimination between the self-elements and the non-self-elements generates mechanisms for the breakdown of immunological tolerance that are harmful to the maintenance of homeostasis (6, 7). Therefore, the organism must have mechanisms to regulate the exacerbated expression of the immune response in order to reduce the harmful effects on the damaged system (8).

In this context, regulatory T cell (Treg) assumes a remarkably important role in regulating the immune response by performing a suppressor function through different mechanisms of action (9, 10). It is not by chance that alterations in the frequency and/or development of these cells are a risk factor for both the susceptibility to infectious agents and the worsening of the clinical condition of an already established pathology (11, 12). One of the best characterized markers of Treg is the forkhead box protein 3 (FOXP3) transcription factor, the production of which is closely related to the development and function of most Treg, both in the subpopulation that develops in the intrathymic environment and in those that are peripherally induced in secondary lymphoid organs (13, 14). Currently, complementary studies on the role of Treg and FOXP3 in a series of pathologies prevail in scientific research, mainly seeking to uncover the therapeutic potential that these factors can offer (15–17).

## FOXP3 Structure

**Figure 1:**
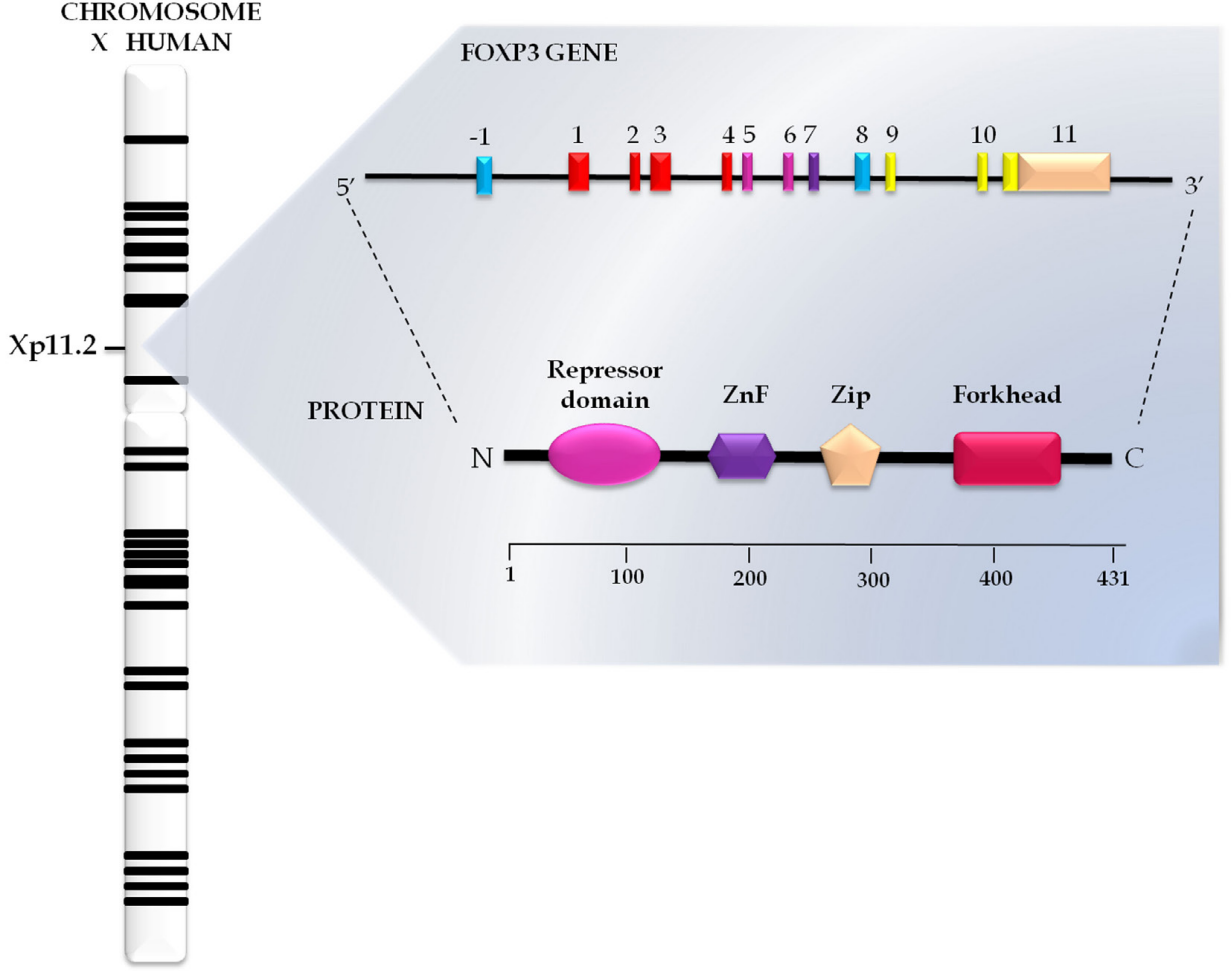
**Genomics and structural organization of forkhead box protein 3 (FOXP3)**. The FOXP3 gene has 11 exons and is located on the short arm of the human X chromosome in Xp11.23 position. This gene encodes a protein FOXP3 with 431 amino acids structurally organized in the repressor domain, the N-terminal portion; zinc finger (ZnF) and leucine zipper (Zip), in the central portion; and forkhead domain, in the C terminal portion.

The transcription factor FOXP3, a 47-kDa protein composed of 431 amino acids (Figure 1), is a member of the subfamily P of the FOX protein family (18). These proteins share a preserved DNA binding domain known as forkhead/winged helix, which contains three α helices and two large loops that resemble the double wings of butterflies (19). More than 100 such proteins have been identified, and a proposal to standardize their nomenclature was developed, resulting in the term FOX (forkhead box); subclasses are indicated with letters and their members with Arabic numerals (“FOX,” subfamily “P,” and member “3”). For humans, the abbreviated name is fully written with uppercase letters (20).

In addition to the forkhead domain located in the C-terminal portion, FOXP3 also has a central domain, which encompasses the C2H2 zinc finger (ZnF) and leucine zipper (LeuZip) regions, and a repressor domain in the N-terminal portion (Figure 1) (21, 22).

Interaction of the forkhead domain with the target sequence of activator protein-1 (AP-1) blocks the activity of the complex formed by AP-1 and the nuclear factor of activated T cells (NFAT), which interferes with T cell activation (23). The ZnF and LeuZip domains are conserved among subfamily P members, being traditionally considered as protein interaction domains responsible for the formation of oligomers essential for the function of FOXP3 (24). In turn, the repressor domain is rich in proline and plays a crucial role in the repression of FOXP3 target genes through its interaction with chromatin remodeling factors (25).

The *FOXP3* gene comprises 11 exons, measures 21 kb and is located on position Xp11.23 in the short arm of the X chromosome (Figure 1) (26). Differential expression of *FOXP3* between species was demonstrated by Western blotting in a study that compared the human and mouse proteins. In humans, FOXP3 occurs in two closely spaced bands, the lower corresponding to the isoform without the second encoding exon (FOXP3Δexon2) and the upper representing the wild isoform, an ortholog of the mouse protein (27, 28). The wild isoform seems to interact with the RAR-related orphan receptor alpha, inhibiting its role as a transcriptional activator (29). In turn, variant isoforms significantly inhibit CD4^+^ T cell activation induced by the chimeric CD28/TCRζ receptor (30).

## FOXP3 Function and Regulation

FOXP3 is an essential molecular marker of Treg development and function in the thymus and peripheral lymphoid organs (31). According to available data, the initial signal for the induction of *FOXP3* expression is triggered by the presentation of peptides derived from host’s autoantigens through T cell receptor–major histocompatibility complex (TCR-MHC) class II interactions (32, 33). The immunostimulation potential of antigens and the early inflammatory environment are determinants of Treg differentiation into new effector phenotypes (34).

Gain-of-function studies have demonstrated a relationship between FOXP3 and Treg. Retroviral FOXP3 transfer to CD4^+^CD25^−^ T cells converted them into a regulatory phenotype similar to the natural lineage; as a result, in addition to ectopic *FOXP3* expression, these cells exhibited low interleukin (IL)-2, IL4, and interferon (IFN)-γ secretion after stimulation and upregulated the expression of typical Treg surface markers, such as *CD25*, cytotoxic T-lymphocyte-associated protein 4 (*CTLA-4*), and *CD103*. These data emphasize the double role of FOXP3 as a transcriptional repressor and activator (31). In its role as a repressor, FOXP3 interacts with transcription factors NFAT (35) and nuclear factor kappa B (NF-kB) and blocks the expression of cytokine-encoding genes (36). However, whether FOXP3 regulates transcriptional activation in a direct or an indirect manner has not yet been elucidated (37).

From the molecular perspective, FOXP3 interacts with the Tip60 histone acetyltransferase and several members of class I and II histone deacetylases. Biochemical and spectrophotometric analyzes reveal that the transcriptional complex of FOXP3 may contain more than 300 associated proteins involved as transcriptional cofactors. This complex regulates chromatin remodeling through histone acetylation and deacetylation and facilitates the interaction of FOXP3 with its target genes (38, 39); it also plays an essential role in the determination of the central and peripheral responsiveness of T cells. The oligomerization status of FOXP3 plays a key role in binding to promoter sequences (40). Interaction between chromatin remodeling complexes may also occur to regulate specific transcription sites located in the same promoter region, thus modulating loci related to cell line identity (41).

Cytokine transforming growth factor (TGF)-β is able to induce *FOXP3* expression and confer functional suppressor activity to T cells initially from a non-regulatory lineage, even in the absence of costimulatory signals. TGF-β also induces secretion of the cytokine IL-10, which is related to the generation of peripheral Treg (pTreg). All together, these data suggest that TGF-β sustains regulatory networks through modulation of *FOXP3* expression and development of ectopic Treg (14, 42, 43). Furthermore, IL-2 sustains the function and survival of Treg through the induction of *FOXP3* mRNA expression and stabilization and the upregulation of pro-survival protein myeloid cell leukemia 1 expression, which counterregulates the pro-apoptotic protein Bim (44). By interacting with TGF-β, IL-2 increases the expression of Treg markers, such as *CD25, CTLA-4, CD122*, and glucocorticoid-induced TNF receptor family related protein (*GITR*) (42). In turn, pro-inflammatory cytokine tumor necrosis factor (TNF)-α might directly influence Treg function through the induction of protein phosphatase 1 (PP1) expression, which dephosphorylates the FOXP3 forkhead domain and changes its transcriptional function (45).

**Figure 2:**
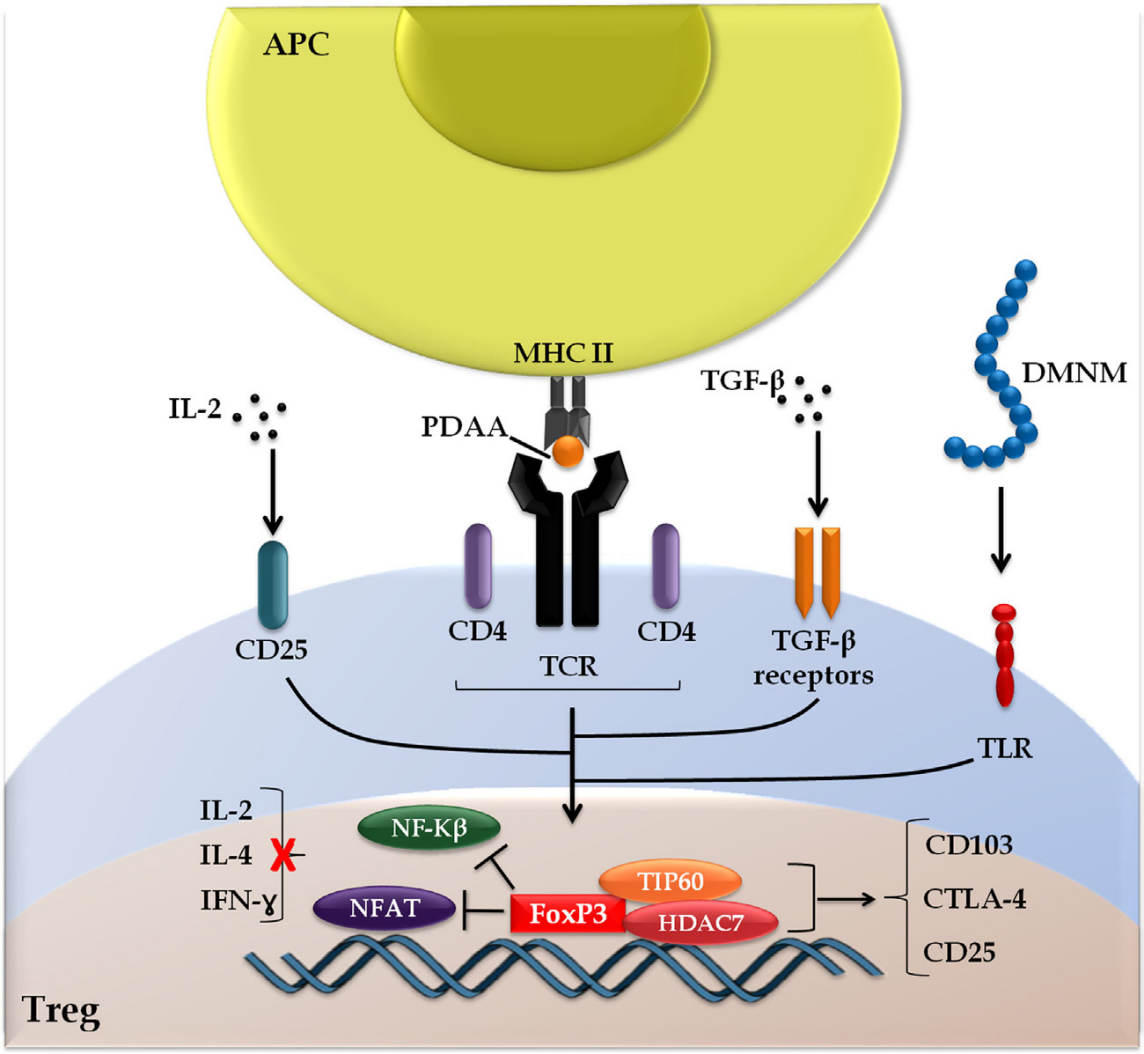
**Forkhead box protein 3 (FOXP3) signaling cascade**. The induction of FOXP3 is initiated following the presentation of peptides derived from autoantigens (PDAA) through the interaction of the T cell receptor with major histocompatibility complex of class II (TCR-MHC II) on antigen-presenting cells (APC). Alternative stimuli include cytokines transforming growth factor (TGF)-β, interleukin (IL)-2, and metabolites derived from the intestinal microbiota (DMNM). FOXP3 interacts with chromatin remodeling factors (TIP60 and HDAC7), which facilitates the dynamics with target genes and prevents the interaction of transcription factors [nuclear factor kappa B (NF-kβ) and nuclear factor of activated T cells (NFAT)] with activating cytokines cell response genes (IL-2, IL-4, and interferon-γ), but promotes the expression of genes linked to regulatory T cell (Treg) activation [CD103, cytotoxic T-lymphocyte-associated protein 4 (CTLA-4), and CD25], highlighting the double FOXP3 function as a transcriptional repressor and activator.

In addition to the central role played by cytokines, Hoeppli and colleagues also addressed other environmental stimuli that influence biological aspects of Treg, such as dietary metabolites, catabolites, and the host microbiota, focusing primarily on their implications for the development of new therapeutic interventions (46). The FOXP3 signaling cascade is summarized schematically in Figure 2.

## Regulatory T Cell

### Phenotypic Diversity

Regulatory T cell is a specialized subset of T cells that induce suppression of the immune response to pathogenic agents as well as non-infectious targets, such as self-antigens and non-noxious external antigens. Thus, Treg maintains self-tolerance through suppression of the activation and expansion of autoreactive cells (47).

**Figure 3:**
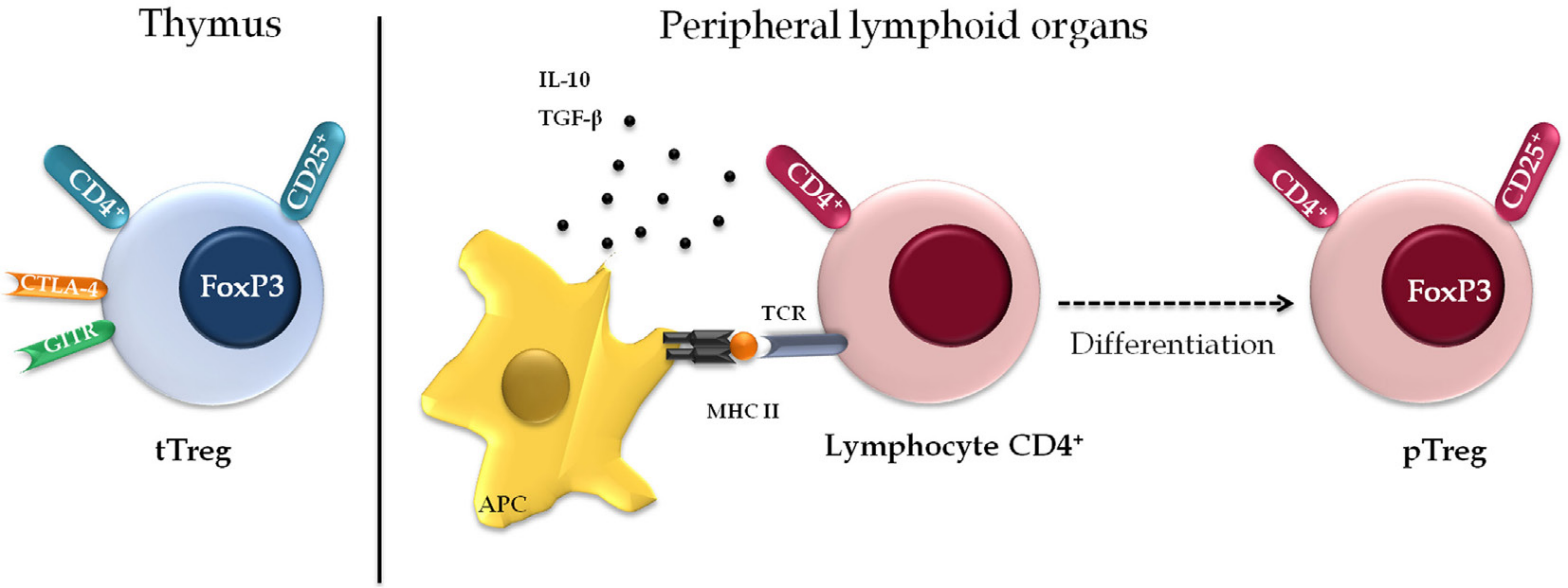
**Phenotypic diversity of regulatory T cell (Treg)**. There are two separate subsets of Treg. The first population of resident cells that is formed along the thymopoiesis and express constitutively markers including CD25, CD4, cytotoxic T-lymphocyte-associated protein 4, and glucocorticoid-induced TNF receptor family related protein. The second subset is formed by a peripheral Treg (pTreg) population that induces regulatory phenotype in the peripheral lymphoid organs, under specific conditions, antigenic stimulus, or suppressor cytokines.

There are two different subsets of Treg (Figure 3). One consists of cells formed along thymopoiesis, resulting in a natural population of resident thymus-derived Treg (tTreg). These cells originate *via* the differentiation of TCR-stimulated naïve T cells or from functionally mature precursors that either do not express the IL-2 receptor α chain (CD25) or lose their ability to express it as a means to keep their suppressor function—although they may express it anew after stimulation by antigens and IL-2, thereby reactivating themselves as Treg (48, 49). Upon generation, these cells migrate to the periphery, where they perform their suppressor function, being essentially costimulated by CD28 to maintain cell survival and homeostasis (50). Most pTreg expresses high levels of *CD25* (*CD25^high^*), a small percentage expresses basal levels of this marker only (*CD25^low^*), and both populations express *FOXP3* (51).

The surface phenotype of tTreg is characterized by constitutive expression of markers *CD4, CD25* (whence they are known as CD4^+^CD25^+^), selectin *CD62L*, integrin *CD103, CTLA-*, and *GITR* (9, 52–54). They might also express protein lymphocyte activation gene 3 (*LAG-3*); TNF receptor 2; and Toll-like receptors (*TLRs*) 4, 5, 7, and 8, among other molecules (54, 55), with differential expression of these markers between humans and mice (56). Basal expression levels of the IL-7 receptor (*CD127*) are characteristic of this cell population, and it is, therefore, used to distinguish them from other lymphocytes (57).

The second subset is composed of so-called pTregs, which are generated in the peripheral lymphoid organs from mature CD4^+^ T cells under certain antigenic stimuli or in the presence of suppressor cytokines. In this population, *CD25* expression varies as a function of the local disease scenario and regulatory activity, and the suppressor ability of these cells is directly cytokine-dependent (9). Some authors have suggested that extrathymic Treg development might also be influenced by cytokine-modified dendritic cells (DCs) able to induce a state of anergy with suppressive properties in T cells (58).

Type 1 Tregs (Tr1) are one of the most common populations of pTreg. They are characterized by significant production of the cytokines IL-10, IFN-γ, IL-15, and TGF-β and low production of IL-4 and IL-2 (59). Anergy and low cell proliferation are attributed to IL-10, which, together with IFN-α, synergistically contributes to Tr1 cell differentiation (60). There is no marker specific for this population, although the repressor of GATA has been suggested as a potential candidate (61).

Th3 cells are the second most frequent population of pTreg. This population originates from TGF-β-stimulated CD4^+^ T cells and plays a central role in oral tolerance to non-self antigens through secretion of IL-10 and TGF-β (62). Other populations that might perform regulatory functions include the CD4^+^Vα14^+^ (NKTreg) (63), CD8^+^CD45RC^low^ (64), γΔ T cells (65), among others.

### Mechanisms of Action

**Figure 4:**
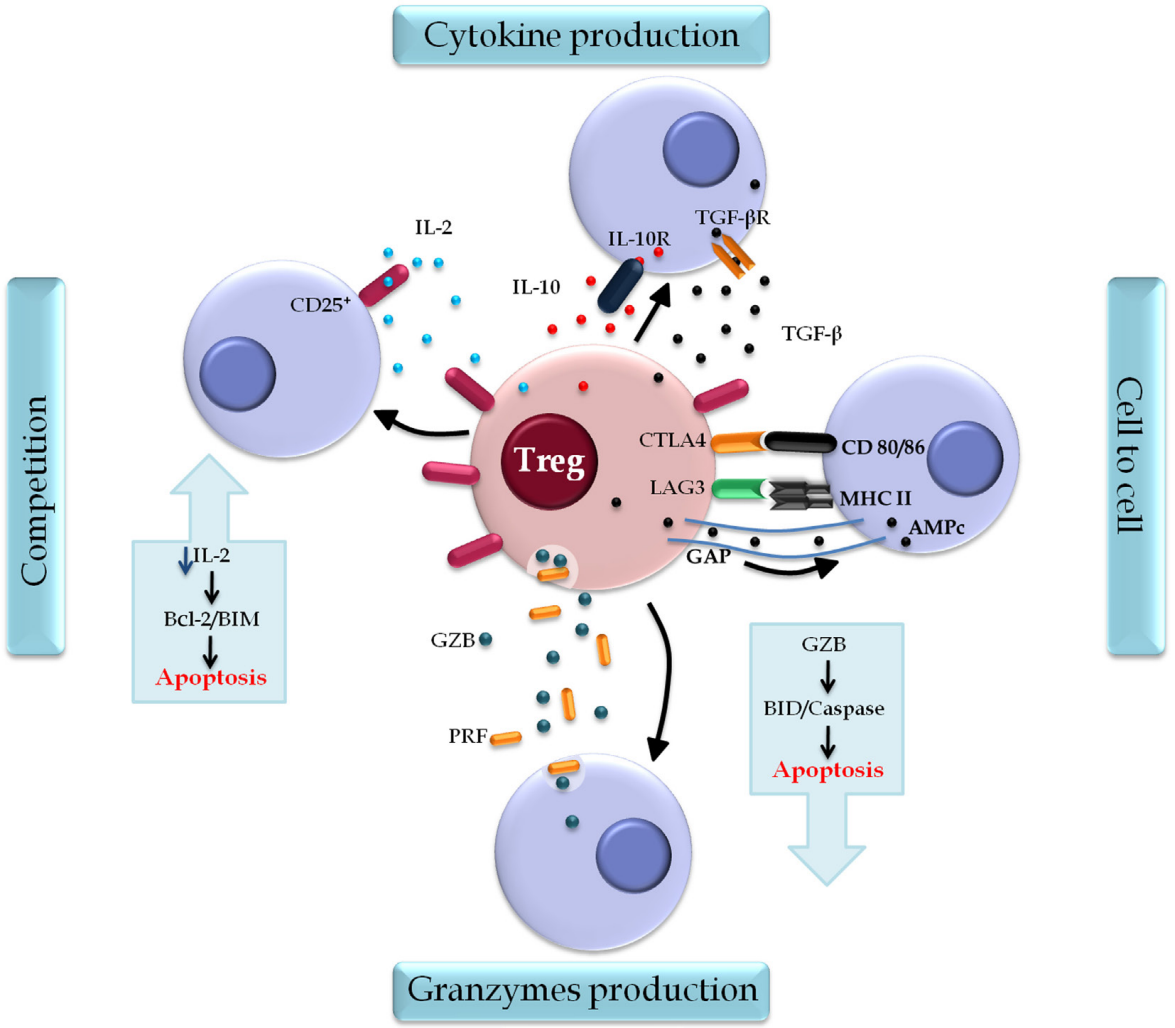
**Regulatory T cell (Treg) action mechanisms**. Tregs induce immune suppression by four classical mechanisms: the contact cell-to-cell is mediated by surface markers cytotoxic T-lymphocyte-associated protein 4 (CTLA-4) and lymphocyte activation gene 3 (LAG-3) that interact, respectively, with molecules CD80/86 and MHC II on the target cells, regulating cell function. AMPc is also a mediator of this mechanism, which is released directly through the gap junctions and inhibits the proliferation and differentiation of the target cell. Cytokine production, in which transforming growth factor (TGF)-β and interleukin (IL)-10 modulate the activation and function of Treg. Competition for growth factors, in particular IL-2, the constitutive expression of CD25 on Treg, and their deprivation induce target cell to apoptosis through the B cell lymphoma 2 protein (Bcl-2)/BIM pathway. A fourth mechanism is found in lineages Type 1 Treg that secrets Granzyme B (GZB) and perforin (PRF) that act specifically in myeloid precursors. The cytolytic activity of GZB in target cells induces apoptosis by caspases or BID pathway.

Regulatory T cells induce immune suppression *via* a set of mechanisms that are clustered into four distinct models: cell–cell contact, cytokine secretion, competition for growth factors, and production of granzyme B (GzB) and perforin (PRF) (66, 67) (Figure 4).

Cell–cell contact is mediated by surface markers, such as CTLA-4 and LAG-3, in addition to other mediators, such as cyclic adenosine monophosphate (cAMP) (66). CTLA-4 regulates the catabolic enzyme indoleamine 2,3-dioxygenase through its interaction with costimulatory molecules CD80/86 present on antigen-presenting cells (APC), which interferes with the activation and proliferation of effector T cells through the reduction of free tryptophan (68). Additional evidence indicates that costimulation by CTLA-4 activates Treg to perform their suppressive action, while such activity is attenuated by a CD28-mediated stimulating pathway. The balance between these two signals might be critical for the determination of the threshold of the immune response (69).

Lymphocyte activation gene 3 was found to modulate Treg function both *in vitro* and *in vivo* even when ectopically expressed (68). Treg might interfere with DC activation *via* interaction of LAG-3 with MHC class II (70); no similar role has been suggested for LAG-3 in the MHC-CD4 interaction despite its superior affinity (71).

Regulatory T cells increase cAMP levels in target cells through two mechanisms: direct release through gap junctions (72) and local adenosine production *via* CD39/73-mediated enzymatic hydrolysis of adenosine triphosphate (73). Interaction of adenosine with the A2A receptor elevates cAMP levels in target cells (74) with consequent inhibition of cell proliferation and differentiation as well as of the expression of cytokines, such as IL-2 and IFN-γ, *via* NF-kB blockade (75).

In addition to modulating Treg activation, secretion of TGF-β and IL-10 is also involved in the maintenance of Treg suppressor activity. TGF-β plays a key role in lymphocyte homeostasis; in Th1 cells, it inhibits the expression of the transcription factor T-bet, thus preventing cell differentiation (76). Similarly, it interferes with the response to IL-2 *via* downregulation of IL-2 receptor β subunit expression (77). TGF-β also influences Th2 cell differentiation through downregulation of the transcription factor GATA-3, a process directly or indirectly dependent on the transcription factor Sox4 (78). IL-10 suppresses T cell responses through downregulation of IL-2, IFN-γ, and granulocyte-macrophage colony stimulation factor (79). In addition, IL-10 modulates B cell function to promote immune tolerance during allergy, which is evidenced through the induction of immunoglobulin IgG4 and the suppression of IgE (80).

By constitutively expressing *CD25*, Tregs are potential competitors for IL-2 relative to their target cells. Lack of growth factors causes apoptosis of target cells both *in vivo* and *in vitro* through the B cell lymphoma 2 protein/Bim pathway and independently of PRF/Fas (81). Changes in suppression might occur following the addition of exogenous IL-2 or reinforcement of its endogenous production *in vitro* (82). Experimental models narrow the relationship of IL-2 to Treg suppressor activity by showing that IL-2 blockade, and consequent loss of stimulation by this pathway, can induce conventional T cells to a stronger cellular response to autoantigens or to environmental antigens (83).

Other authors have emphasized an additional mechanism of suppression in Tr1 lines characterized by cytolytic activity on target cells of myeloid origin and mediated by GzB and PRF and dependent on MHC class I, CD54, CD58, CD155, and CD122. The GZB induces target cell apoptosis *via* caspase or *via* BID (67).

### Treg: Immune Tolerance and Suppressive Response

The first relevant steps toward the elucidation of the immune tolerance were made at the turn of the 1970s. First, Nishizuka and Sakakura reported in 1969 the occurrence of autoimmune disorders in young normal mice subjected to thymectomy. Then, in 1973, Penhale and colleagues found that normal adult mice subjected to thymectomy and irradiation developed autoimmune thyroiditis (84, 85). Several interpretations were suggested for these phenomena, one of which addressed the alteration of T cell homeostasis, leading to uncontrolled proliferation of autoreactive cells (86). However, the following series of studies indicated that Treg depletion was a plausible mechanism for loss of immune tolerance and consequent development of chronic autoimmune diseases (87, 88).

After the association of mutations in *Foxp3* with the immunological status of the mutant strain scurfy (sf), it was possible to discuss the relevance of genetic mechanisms in the control of immunological homeostasis (89). Subsequently, studies reported the importance of *Foxp3* gene expression in the development and function of Treg both in the thymus and peripherally (13, 90). Therefore, Treg plays an essential role in the suppression of the immune response to a wide variety of self- (autoimmune response) and non-self-antigens and needs to be further investigated as a specific therapy for immune tolerance (91, 92).

Regulatory T cell is involved in immune phenomena occurring in a wide variety of organs. In a mouse model of liver injury induced by concanavalin A (Con A), a mitogenic lectin, tolerance was due to increased IL-10 production by Treg (93). In a later study, the frequency of Treg in the liver increased after Con A injection, with this population being phenotypically different from that in the spleen, as it characteristically exhibited increased *Foxp3, CTLA-4, GITR*, and *CD103* expression. The authors further suggested that Treg activity depended on TGF-β because blockade of TGF-β signaling increased susceptibility to liver injury (94).

The intestinal microenvironment is a preferential site for development of Treg induced by microbiota-derived antigens. Suppression of the immune response has effects on local homeostasis, as any breakdown of the Treg network causes chronic inflammation (95). In this context, IL-23 is suggested as a limiting factor of the regulatory mechanisms of Treg from the intestinal inflammatory response. The IL-33/ST2 pathway activation is notorious for the adaptation of Treg to inflamed tissue and maintenance of immune regulation (96), in fact, blockade of the ST2 pathway may affect the activation of Treg, as shown in mice with insulin resistance associated with obesity, which thus affects the adipoimmune profile in the pathological condition (97). However, studies show that the transferred Foxp3^+^ or CD45RB^low^ and CD45RB^low^CD25^+^ Treg with regulatory potential were sufficient to prevent Th1- and Th17-mediated intestinal inflammation, as shown in a model of colitis in mice (98).

Regulatory T cell also participates in the maintenance of skin immune homeostasis, representing 10% of all T cells in the normal skin. The expression of E- and P-selectin ligands (*E-/P-Lig*) is indicative of Treg kinetics across the epithelial tissue. Constitutive recirculation of Treg through the skin is severely impaired in the absence of the enzyme FuT7, and blocking Treg migration to the skin results in severe tissue-specific inflammatory disease (99). Memory Treg (mTreg) is maintained in the tissue for long periods, even post antigenic stimulus, but its suppressive capacity is increased when the antigen is expressed again, thus limiting the severity of inflammatory or autoimmune reactions in the tissue. Treg naturally passes through the phases of generation, proliferation, and differentiation in specific memory populations, which express high levels of activated Treg markers, but with low expression of *IL-2* and *IL-7*, indicating them as a final stage of differentiation of Treg (100–102).

In the oral mucosa, Treg conversion is induced by antigens and reinforced by TGF-β and retinoic acid. The reinforcement by retinoic acid results in up-regulation of integrin α4β7 and chemokine CCR9, whereby Treg accumulates in the gut-associated lymphoid tissue, with cytokine secretion being one of the main mechanisms of maintaining oral tolerance, in particular IL-10 and TGF-β (103). The oral Treg population is characterized by expression of latency-associated peptide, which is non-covalently associated with the amino-terminal domain of TGF-β, forming a latent TGF-β complex (104). Although also induced during oral tolerance and mainly related to the suppression of the Th1/Th17 response, the CD8^+^CD45RC^low^ Treg subpopulation (64) is less prevalent in the oral mucosa than CD4^+^ cells (105, 106).

Molecular abnormalities in autoreactive Treg might trigger resistance to Treg-mediated suppression and the breakdown of tolerance. One study found that hyperreactive T cells of mice lacking Clb-b, a protein that participates in CD28 activation, were resistant to Treg-mediated suppression in an *in vitro* model due to increased secretion of IL-2 following TCR stimulation, which neutralizes Treg-mediated suppression (107).

### Immunoregulatory Role of Treg in Transplants

The immune response plays a key role in graft rejection; for this reason, much effort has been devoted to the development of protocols to induce immune tolerance in this setting (108). Tregs stand out because their primary and homeostatic regulatory mechanisms are necessary to prevent immune graft rejection (109).

In experimental models, the high frequency of CD4^+^FOXP3^+^ Treg exhibited by recipients of allogeneic bone-marrow transplants was found to be inversely proportional to the incidence of deaths by acute graft-versus-host disease (GVHD), which emphasizes the role of Treg as mediators of immune attenuation. The prognostic value of Treg at the onset of GVHD is directly related to the improvement of the clinical status of patients; however, the use of immunotherapy agents, such as calcineurin inhibitors, might impair Treg expansion and function (110–112).

Al-Wedai and colleagues found that the frequency of Treg was significantly lower in patients with chronic rejection compared to stable kidney transplants, which emphasizes the role of Treg in local immune tolerance (15). In a model of adoptive Treg transfer between mice, the number of Tregs gradually increased in the recipients following injection of allogeneic Treg every 2 weeks. This finding lends support to the hypothesis that Treg generated *ex vivo* can act as a therapeutic vaccine to induce suppression in the host (113). While the frequency of Treg after transplantation decreases over time, the suppressive capacity of this cell population remains unchanged or increases (16).

Different Treg populations with different transient roles and in specific concentrations are likely needed for the maintenance of transplantation tolerance. The coexistence of natural and Tr1 was demonstrated in a mouse model of induced tolerance after pancreatic islet transplantation. While the frequency of tTreg was significantly higher at the onset of graft rejection and then returned to physiological levels soon after, the frequency of Tr1 cells remained high over a long period of time. Differences in location and migration, duration of action after transplantation, and maintenance of long-term tolerance were also observed between these cell populations (114, 115).

Stimulation with IFN-γ generates conditioned alloreactive Tregs, which are phenotypically characterized by *CD25, CD62L*, and *FOXP3* expression. In addition, they inhibit skin graft rejection and vasculopathy associated with posttransplant arteriosclerosis by controlling reactive T cell infiltration into the transplanted organ. *In vitro* production of these cells might result in clinical benefits *via* T cell regulation after transplantation (116–118).

Immunosuppressive drugs are used to reduce the occurrence of episodes of graft rejection. Rapamycin (also known as sirolimus), a macrolide antibiotic that inhibits cell cycle progression in T cells, might induce conversion of peripheral CD4^+^CD25^−^ T cells to CD4^+^FOXP3^+^ Tregs potentially able to suppress effector T cell proliferation while maintaining their antigenic specificity (119). Sirolimus favorably regulates the Th17/Treg axis; this was demonstrated by suppression of Th17 and upregulation of Treg, which contribute to kidney graft acceptance (120). Clinical data point to the improvement of chronic nephropathy and long-term kidney function as well as to the reduction of vascular abnormalities in patients given sirolimus as an immunosuppressant (121).

Calcineurin inhibitors significantly reduce the occurrence of acute episodes of kidney graft rejection compared to azathioprine (122) [a synthetic purine analog with immunosuppressive properties (123)]; however, they cause considerable long-term side effects, including nephrotoxicity and graft failure (124). Efforts are currently devoted to the identification of markers of graft acceptance likely to contribute to minimize immunosuppression. The role of the mTreg/Treg balance in the dynamics of treatment is one of the main issues requiring elucidation (125).

## Relevance of FOXP3 and Treg in Pathological Conditions

### Autoimmune Diseases

**Figure 5:**
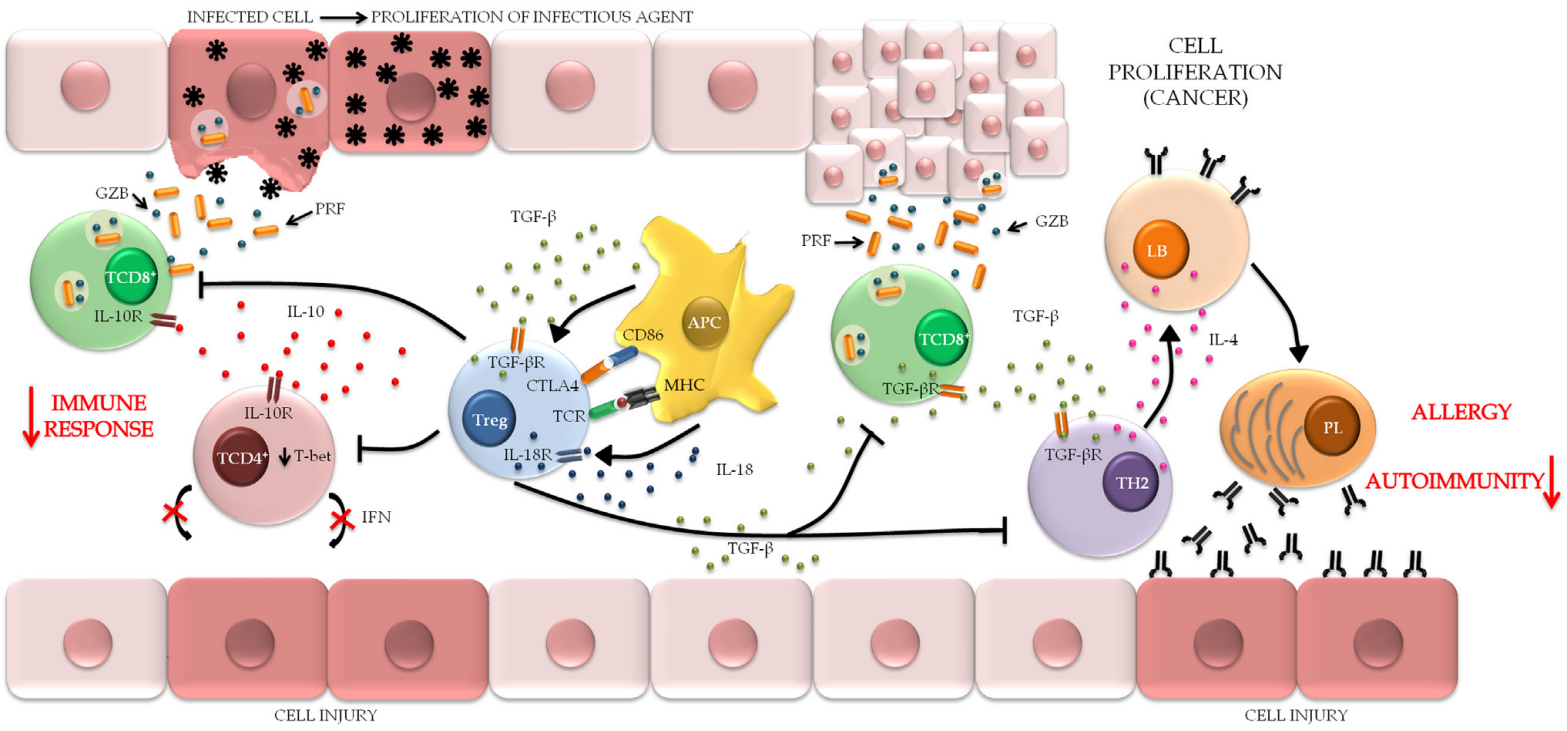
**The role of regulatory T cell (Treg) in maintaining the pathological condition**. Treg can be stimulated by different mechanisms through the interaction with APC. When activated, Treg attenuates the harmful immunological responses usually associated with chronic inflammatory conditions, autoimmune diseases, and allergic processes of the mucosa, in order to maintain immune homeostasis and decreased tissue injury. In contrast, the maintenance of the immune response provides the persistence of infectious agents and favors the establishment of microenvironments that are conducive to tumor metastasis (TCD8^+^, cytotoxic T lymphocytes; TCD4^+^, helper T lymphocytes; APC, antigen-presenting cells; TH2, T helper type 2 lymphocytes; LB, B lymphocytes; PL, plasmocytes).

As Tregs are clearly associated with the maintenance of immune homeostasis, one may safely assert that, in autoimmune diseases, these specialized cells participate in complex regulatory mechanisms aimed at recovering the natural pattern of immunity (Figure 5).

In a model of transgenic mice (named SFZ70) reactive to skin autoantigens, TCR-autoantigen stimulation induced the development of active Treg able to migrate to the skin and inhibit the expression of tissue-homing molecules and pro-inflammatory cytokines by conventional T cells (126). Thus, one might assert that the reverse is also true, i.e., abnormalities in the function of different Treg populations contribute to the genesis of autoimmune diseases. For instances, Treg function is significantly reduced in patients with multiple sclerosis compared to healthy individuals (127) as well as the reduction of *FOXP3* expression (128). When stratified for the severity of sclerosis, an inverse relationship is suggested between the frequency of Treg and the disease score (129), indicating that the loss of immune tolerance may contribute to the emergence of autoimmunity against components of the central nervous system, although the frequency of these cells does not change significantly when compared to healthy individuals, or between the cerebrospinal fluid and the peripheral blood of these patients (12). The proportion of Treg reflects on clinically evident sexual dimorphism in the incidence of sclerosis, in which women show a higher frequency of these cells in cerebrospinal fluid (130). Therapeutic regimens have distinct functional effects on the Treg population, whereas fingolimod treatment does not alter the frequency of these cells (131), maintenance of anti-CD25 monotherapy substantially reduces the frequency of Treg without altering tolerance in the central nervous system (132).

The conclusion about the frequency of Treg in rheumatoid arthritis (RA) remains conflicting. While studies report a significant increase in Treg in peripheral blood and synovial fluid of RA patients (133), other studies show a decrease in the proportion of these cells in patients’ blood circulation (134). The discrepancy of these results may be related to the diversity of Treg phenotypes that manifest distinctly depending on the stage of the disease: there is a decrease in the CD4^+^ CD25^high^ phenotype in the early stage of the disease (135), while the CD4^+^ CD25^+^ FOXP3^+^ is not altered in the inactive phase (136), and the percentage of Treg cells expressing the CD45RO memory phenotype increases both synovial fluid and peripheral blood independent of the degree of disease activity (137), indicating that some functional Treg phenotypes preserve regulatory activity as a way to mitigate collateral damage, although, in a general context, an exacerbated immune response prevails in RA (138). In this context, treatment with TNF-suppressing drugs, such as infliximab, helps in the recovery of Treg number and functionality (139). Research also points out that histone deacetylase inhibitor drugs may induce *FOXP3* expression and the suppressive capacity of Treg *in vitro*, suggesting that these drugs may in future be included in therapeutic regimens for autoimmune and inflammatory diseases (140).

Evidence shows that the reduction of Treg frequency is one of the factors related to the clinical manifestations of systemic lupus erythematosus (SLE) (141), and the suppressive function of these cells depends on the stage of the disease (142), as demonstrated in thrombotic thrombocytopenic purpura associated with SLE, whose CD4^+^CD25^+^ phenotype was correlated with disease severity (143); and in patients with lupus nephritis, where the high expression of *FOXP3* was associated with the systemic and histological activity of the disease and could be used in the risk stratification of the disease, suggesting FOXP3 as an analytical biomarker of autoimmune activity (144). Other studies, however, show that the number of Tregs increases in SLE as a mechanism contrary to the establishment of the pro-inflammatory condition (145). New therapeutic platforms are being explored in order to aid in the functional reestablishment of Treg in lupus. In double knockout models for DEF-6 and SWAP-70, it is proposed that both factors represent important regulatory proteins that maximize the ability of Treg to cope with the chronic inflammatory condition (146). As well, mesenchymal stem cells can produce immune tolerance-inducing molecules, such as TGF-β, both *in vivo* and *in vitro*, which highlights the potential of these cells as a future therapeutic scheme (147).

Forkhead box protein 3 was suggested as an analytical biomarker of autoimmune activity. In patients with lupus nephritis, high *FOXP3* expression was associated with systemic and histological disease activity and could therefore be used for risk stratification (144). The literature points to mutations in FOXP3 as the main genetic factor that interferes with Treg function; however, one patient with non-immunodeficiency, polyendocrinopathy, and enteropathy X-linked (IPEX) autoimmune enteropathy had no mutations in the FOXP3 locus and did not exhibit chronic reduction of Treg in the bloodstream or accumulation of FOXP3^+^ Treg in the inflamed duodenum during the active stage of disease, which could have contributed to the loss of local immune control. Global lymphocytopenia affecting various T cell populations was due to defective production and export of thymic cells, which lends support to the hypothesis that the disease has an autosomal pattern of inheritance (148).

The fact that the liver microenvironment induces immune tolerance partly *via* generation of Treg leads one to question the possible participation of these cells in autoimmune hepatitis (AIH). Selective autoantigen stimulation is known to lead to Treg generation in sinusoidal endothelial cells *in vivo*, which is considered to be an effective anti-autoimmune mechanism (149). In a mouse model of primary biliary cirrhosis, which is similar to the human disease, sf mice were used to demonstrate that abnormal Treg function is accompanied by exacerbation of CD8^+^ T cell-mediated bile duct autoreactive damage (150). In human AIH, although numerically and functionally abnormal, Tregs reduce the production of Th1 cytokines and promote secretion of Th2/Th3 cytokines *via* cell–cell contact (151). Reduction of Treg might occur in response to treatment with steroids/azathioprine, which are the recommended drugs because they preserve immune regulation and promote long-term control of AIH (152, 153). However, some studies with discrepant results have suggested that the frequency and function of Treg are not affected in AIH, although they might be correlated with disease severity. AIH progression might be due to resistance to Treg activity, inflammatory stimuli too strong for the intrahepatic Treg, or lack of identification of liver autoantigens, with consequent absence of tissue suppression (154).

### Allergies

In the traditional model of IgE-mediated allergic responses, imbalance of the Th2 profile triggers chronic inflammation typical of the acute response on skin or mucosal surfaces, and deviations in this proportion distinguish healthy and allergic responses (155) (Figure 5). In a mouse model of atopic dermatitis, increased expression of Th2 cytokines and elevated IgE levels exhibited inverse correlations with Treg depletion (156). Similar phenomena were detected during the sensitization phase in a murine model of experimental allergic airway inflammation, which suggests that Treg-mediated maintenance of tolerance against allergens occurs spontaneously as early as the sensitization phase (157). In humans, the proportion of FOXP3^+^CD25^high^ Treg in umbilical cord blood and early childhood exhibited positive correlations with sensitization at the age of 18 and 36 months. Nevertheless, more complex studies are needed to confirm whether the Treg proportion precedes sensitization and whether high levels of Treg might interfere with the natural development of T cells during childhood (158). Regarding seasonal allergy in humans, the presence of FOXP3^+^ Treg in the nasal mucosa after immunotherapy is associated with the clinical efficacy of treatment (159).

Based on the functional characteristics of Tr1 cells, some authors have asserted that Th2 regulation depends on the secretion of IL-10 and TGF-β and expression of *CTLA-4* and programmed death-ligand 1 (155). However, no significant differences were found in TGF-β expression between children with and without food allergies, which might be considered a phenotypic singularity of Treg specifically involved in this type of allergy (160). Singular phenomena were also detected in patients subjected to allergen-specific immunotherapy, as the frequency of Treg did not depend on the nature of the allergens but rather on the degree of severity of clinical symptoms and the specific location of the allergic reaction (161).

### Cancer

Induction of Treg within the tumor microenvironment has been suggested as a mechanism likely to cause impaired anticancer immunity, and the expansion of this cell population might be considered a true hindrance to the efficacy of immunotherapy (162) (Figure 5). Radiation-induced immunosuppression is an example of Treg induction giving rise to a microenvironment both susceptible to and permissive for cancer and mutagenesis (163).

Studies have shown an association between Treg infiltration into the liver and recurrence of tumor metastases and multicentric cancer after hepatic resection (164), coinfiltration by FOXP3^+^ Treg and B7-H1^+^PD-1^+^ T lymphocytes with high-risk breast cancer (165), density of FOXP3^+^ lymphocytes with lymph node metastases of pancreatic cancer (166), and expression of *FOXP3* and *p16^INK4a^* with progression of cervical cancer, more specifically with lymph node metastasis (167). Divergences on the prognostic value of Treg were discussed in the meta-analysis of Zhao and colleagues. Upon ignoring the possible combinations of various Treg markers, these authors concluded that Treg infiltration into the lung tissue did not affect the survival, relapse, or global survival rates of patients with non-small-cell lung cancer. However, inclusion of studies that analyzed FOXP3 alone or in combination with other markers showed that global survival was significantly associated with cancer (168). New therapies based on reducing or inhibiting *FOXP3* expression to reduce abnormal Treg proliferation stabilize the tumor microenvironment and block the immune escape of tumors, and consequently metastasis needs to be discussed (169).

Other Treg subpopulations might also be induced within the tumor microenvironment. The human ovarian carcinoma SK-OV-3/A2780 cell line is able to convert *in vitro* CD8^+^ T cells into a CD8^+^ Treg phenotype that promotes tumor progression through immune regulation partially mediated by TGF-β1 and IFN-γ (170).

In molecular terms, signet ring cell carcinoma cells might assume functions similar to those of Treg, as evidenced by *FOXP3* expression, which enables them to escape immune surveillance and induce lymph node metastases (171). In stomach cancer, hypoxia potentiates Treg-mediated immune escape through the regulation of cytokines, TGF-β1 in particular (172). The neuropilin 1 (NRP1) is suggested to be another marker correlated with Treg carcinogen maintenance. Vascular endothelial growth factor produced by the tumor induces the migration of Treg NRP1+ to the intratumoral environment, resulting in a local immunosuppression favorable to tumor growth (173). Additionally, the cofactor semaphorin-4a interacts with NRP1 activating the threonine serine protein kinase B/phosphatase and tensin homolog phosphorylation pathway, which assists in maintaining the cell stability of intratumoral Treg (174).

However, the *FOXP3* gene may also act as a tumor suppressor in cancer models. In breast cancer, for example, both control of *HER-2/ErbB2* oncogenes expression and the marked frequency of mutant isoforms of *FOXP3* indicate that FOXP3 is an important suppressor of the carcinogenic condition (175); in a study in which 68.5% of the patients with prostate cancer expressed *FOXP3, FOXP3* behaved as a tumor suppressor. According to the authors, these findings suggest that suppression of malignant transformation is due to FOXP3 repression of *c-MYC*, an oncogene traditionally expressed in tumors (176).

### Cardiovascular Diseases

Atherosclerosis is a chronic inflammatory cardiovascular disease that begins with infiltration of immune cells, which contributes to the development of typical lesions (177). Within this context, Tregs stand out as an intrinsic atheroprotective mechanism due to their ability to suppress the inflammatory response, as was discussed above (Figure 5).

Experimental studies with mice have found that Treg transfer hinders the development of atherosclerotic plaques and, reciprocally, that Treg depletion is associated with worsening of lesions (178). Studies with chimeric (DEREG/*Ldlr*^−/−^) mice both confirmed the relationship between Treg and atherosclerosis and also showed that depletion of these cells worsened hypercholesterolemia in the experimental animals, thus emphasizing the impact of immune mechanisms on metabolic events (179). Administration of IL-2 considerably increased the frequency of Treg and suppressed the IL-10-dependent Th1/2 effector response, with a 39% reduction of atherosclerotic plaque formation. While treatment did not induce regression of preexisting plaques, it significantly enhanced lesion stabilization (180).

Mor and colleagues found that Tregs were significantly lower in patients with acute coronary syndromes compared to patients with stable angina and healthy controls. Curiously, the Treg number was similar between the latter groups, albeit with comparatively different suppressor functions (181). Similar phenomena have been described for atherosclerotic plaques, although the frequency of Treg was higher among patients with more advanced lesions (182).

Downregulation of *CD1a* (of DCs) and *FOXP3* occurs in different clinical subsets of coronary artery disease (183). The tolerogenic profile resulting from Treg (CTLA-4) and DC (CD86) interactions indeed increases atheroprotective effects *via* cell–cell contact (184).

### Viral Infections

Models based on the transfer or removal of Treg from hosts allow measurement and evaluation of their roles in the pathogenesis of viral infections (185). Using such methods, it was recognized that Tregs interact with and modulate antiviral response pathways, particularly in the case of chronic and persistent infections (Figure 5); the range of mechanisms through which viruses trigger Treg responses remains a subject of current debate (186). Modulatory molecular mechanisms have been described for herpes simplex virus type 1 (HSV-1) infection; interactions between viral glycoprotein HSVgD and herpes virus entry mediator (HVEM) were found to promote Treg proliferation; in turn, preactivated Tregs upregulate HVEM, inducing positive feedback between both (187), although other authors argue that Treg may unexpectedly facilitate early immune responses in HSV-infected mice by orchestrating homing of effector cells to the site of infection (188). Methyltransferase SMYD3 was suggested as a primary epigenetic marker of FOXP3 regulation in mice infected with respiratory syncytial virus (189). Upon infection with the porcine reproductive and respiratory syndrome virus (PRRSV), high Treg frequency in association with viremia emerged as a promising parameter for future studies of the immunobiology of PRRSV infection, although no specific molecular pathway has been established (190). However, in infection with rotavirus, Tregs were not found to have an impact on rotavirus clearance or specific antibody levels, although the hypothesis that Treg depletion might interfere with IgA titers at the peak of disease cannot be ruled out (191).

Regulatory T cells are generated intrathymically in response to a peptide derived from the influenza virus hemagglutinin and then undergo functional specialization along the course of the immune response (192). In the early stages of infection, influenza virus induces a massive regulatory response characterized in *ex vivo* models by robust Treg suppressor activity preceding the establishment of adaptive immunity (193). In aged mice, for instance, a high frequency of tTreg interfered with the primary immune response to flu (194). The data available for secondary infections support the hypothesis that there are antigen-specific mTregs that regulate the immune response and limit the immunopathology of disease to a degree similar to that of primary infection (195).

Controversial findings indicate that, rather than suppressing, Tregs indirectly promote B cell and T follicular helper (T_FH_) responses to influenza through reduced availability of IL-2 (196). Immunization of BALB/c mice with a prototype of inactivated influenza PR8/A/34 virus vaccine expanded the Treg pool but had no effect on the specific B cell response, although it effectively suppressed helper and memory T cells (Tmem) induced by vaccination (197).

The increased frequency of Treg found in infection with the human T-lymphotropic virus 1 (HTLV-1) lends support to the alleged advantage of these cells as hosts, as they contribute to viral expansion due to their hyperproliferative profile, while their immunosuppressive potential enables them to play a pivotal role in immune escape (198, 199).

Data on adult T cell leukemia indicate that some leukemia cells might adopt a suppressor profile similar to that of Treg when originating from FOXP3^+^ precursors (200). The low frequency of Treg in HTLV-1-associated infective dermatitis is related to the maintenance of a microenvironment permissive for lymphocyte expansion and establishment of typical lesions (201). Reduced *CTLA-4* and *GITR* expression in Treg might influence the paradoxical state defined by high Treg frequency with persistence of the inflammatory profile, which is characteristic of tropical spastic paraparesis (also known as HTLV-1-associated myelopathy) (199). Potential dysfunction of Treg in human immunodeficiency virus (HIV)/HTLV-1 coinfection-associated neuropathy is due to transient *FOXP3* expression, which does not convey regulatory properties (202). Such a complex scenario is determined by viral regulatory proteins. HTVL-1 basic zipper factor, for instance, might interact with the SMAD 2/3 and P300 pathways, which results in increased *FOXP3* expression (203). However, Tax causes a reduction in *FOXP3* expression through inhibition of the TGF-β/SMAD pathway (204) and demethylation of the Treg-specific demethylated region gene (205).

Regulatory T cells are cell reservoirs for HIV; their suppressive capacity is not influenced by the virus, as the inhibition of CD4^+^ T cells is preserved and might contribute to virus propagation in the lymph nodes (206). Follicular Treg cells (T_FR_) help in this process because their expansion into secondary lymphoid tissues is related to the inhibition of T_FH_ proliferation and IL-4 and IL-21 production in infections caused by HIV and simian immunodeficiency virus (207). Data suggest a dominant mechanism of suppression by Treg that might reduce *in vivo* antiviral responses that participate in the inability to eradicate HIV infection; however, *ex vivo* studies have not been able to corroborate this hypothesis (208). Reductions in *FOXP3* methylation levels were detected in patients undergoing antiviral therapy, which altered gene expression and increased Treg infiltration of the colonic mucosa (209). However, other studies did not find correlations between FOXP3 expression and the highly active antiretroviral therapy (210); thus, the participation of Treg in therapeutic interventions remains controversial.

The fact that some patients with acute hepatitis B virus (HBV) develop persistent infection associated with T cell hyporeactivity and dysfunction suggests a contribution of Treg in this infection (211). The frequency of Treg fluctuates along the course of acute infection, and the cells exhibit greater suppressor activity against HBV-specific T cells compared to non-specific responses (212), although they do not seem to influence the development of immunological memory (213). High Treg frequency was also reported in the blood and liver tissue of individuals with chronic HBV infection, as was increased proliferation of HBV-specific T cells after the Treg frequency decreases. These cells are directly related to the envelope antigen (HBeAg) and TGF-β serologic status, and their distribution across the body varies according to the clinical status of patients (212, 214, 215).

In hepatitis C virus (HCV) infection, Tregs are associated with viral persistence and weak T cell responses (216). Studies investigating the relationship of Treg with hepatitis C have emphasized one particular role played by the various Treg populations in the pathogenesis of infection, which is especially relevant for the host’s protection against tissue damage (217–219). Interestingly, in one study, HCV-infected patients with normal alanine aminotransferase levels exhibited higher frequency of HCV-specific TGF-β-producing Treg and reduced liver inflammation (220). *FOXP3* expression was found to be elevated in patients with liver cirrhosis and was associated with the expression of other immunogenetic markers, such as *FAS* and its receptor *FASL* (221).

### Bacterial Infections

The role of Treg in the immune response is considered contradictory, in as much as they may facilitate pathogen persistence by regulating the suppression of effector responses potentially noxious for tissues (222) (Figure 5). As an example, the frequency of tTreg significantly increased in the blood and spleen of mice subjected to experimental sepsis, which was likely correlated with *IL-6* expression (223). Furthermore, expression of *TLRs* in Treg is indicative of an additional molecular mechanism for the maintenance of immune regulation (224). Attempts at reestablishing immune homeostasis, in sepsis notwithstanding, indicate that regulatory mechanisms are not restricted to the pathological condition and thus contribute to immune dysfunction as well as the progression of disease (225).

The participation of Treg in mycobacterial infections has been analyzed. In patients with active tuberculosis, increased frequency of Treg was found to be associated with suppression of IFN-γ by Th1 cells, with consequent reduction of tissue damage (226). FOXP3^low^ Treg inhibited the growth of *Mycobacterium tuberculosis* in human macrophages and mice through production of a rho GPD dissociation inhibitor (D4GDI), thus contributing to the development of effective immunity against the pathogen (227). Holla and colleagues found that SHH-PI3K-mTOR-NF-kB signaling in DCs was necessary for *Mycobacterium bovis* BCG-specific pTreg expansion, while NOTCH1 signaling hindered the ability of infected DCs to expand Treg. As a result, these authors emphasize the ability of mycobacteria to regulate signaling molecules involved in the immune response, consequently determining the latter’s functional direction (228).

*Helicobacter pylori* exerts a similar pressure on DC maturation, resulting in their conversion to the semimature and tolerogenic phenotype CD11c^+^MHCII^hi^CD80^lo^CD86^lo^; this subset is unable to activate the effector functions of naïve T cells but is an efficient inducer of FOXP3^+^ Treg in a cell contact-, IL-18-, and TGF-β-dependent manner (229). *H. pylori*-specific Tregs were found to suppress the inflammatory response and reduce gastric ulceration (230). FOXP3 and cAMP were found to upregulate the expression of microRNA *miR-155* both *in vivo* and *in vitro*, which might represent a new infection-associated carcinogenic mechanism (231). Reductions of Treg frequency had impacts on the tolerance of mice infected as neonates, resulting in significant reductions in bacterial load and development of immune disorders, similar to the pre-neoplastic lesions typically exhibited by adult-infected mice (232).

Regarding commensal microbiota, the role of gut bacteria in regulation of the immune response against gastrointestinal pathogens and the maintenance of homeostasis is currently being assessed (233). Continual exposure to gut bacterial antigens was crucial for the induction of CD4^+^CD62L^+^GITR^+^IL-10^+^ Treg, which exhibited protective behavior in a transfer model of colitis in mice (234). Commensal *Clostridium* strains were able to induce Treg in mice and humans (235); clusters IV and XIVa colonizing the colon of mouse pTreg through pathways independent of TLRs, NODs, and Dectin-1 (236). Furthermore, metabolites of commensal bacteria might regulate Treg development. For instance, short-chain fatty acids reacquired through microbiota reconstitution restored the number of Treg in germ-free (GF) mice (237). Butyrate enhanced acetylation in the *FOXP3* locus, resulting in epigenetic regulation of its expression (233, 238). Propionate potentiates this phenomenon by mediating the extrathymic differentiation of Treg dependent on CNS1, a specific intronic enhancer (239). However, in the models of GF mice and specific pathogen-free, it is shown that the development and function of Treg occur independently of the presence of the commensal microbiota, probably restricted to the expression of cytokines and costimulatory molecules, which succumbed to discussions about the real role of the microbiota in immunological homeostasis (240).

### Fungal Infections

Continuous contact of immunocompetent hosts with fungal antigens, either as spores spread in the air or through interaction with the microbiota, does not seemingly trigger hypersensitivity reactions or actual fungal infections. This fact denotes the existence of an effective protective immune response and/or regulatory mechanisms that modulate inadequate immune reactions (241). In the latter scenario, Tregs have been suggested as an essential component of antifungal tolerance.

The expression of Treg markers was higher in patients with lobomycosis compared to the control group. This finding lends support to the hypothesis that Tregs play a dominant role in the lesion microenvironment, limiting the polarization of the Th17 response and hindering the development of an effective response (242, 243). Similarly, in patients with paracoccidioidomycosis skin lesions, Tregs predominate over other T cell subsets, resulting in control of disease progression. One study emphasized the high frequency of FOXP3^+^ Treg in lesions with compact granulomas as a possible mechanism for chronicity (244). In mice infected with *Paracoccidioides brasiliensis*, anti-CD25 treatment caused early Treg depletion, resulting in less severe infection; migration of effector cells to the site of infection was restored, interrupting the progression of infection (245).

A comparison of *Candida albicans* and *Aspergillus fumigatus* infections demonstrated an increase in the Treg/Tmem ratio in the latter, which favors immune suppression (246). Epitopes derived from *A. fumigatus* are able to suppress the expansion of effector T cells and maintain antifungal immune homeostasis through Tr1 cell stimulation (247). *In vitro* stimulation of DCs with *A. fumigatus* conidia induced expression of Th1- and Treg-related cytokines but was a weak inducer of Th2/17 responses (248).

Concerning yeast-like fungi, the study of Schulze and colleagues (249) showed that the frequency of Treg in the lungs significantly increased during the first weeks of infection with *Cryptococcus neoformans* (likely through a TGF-β-dependent pathway), with suppression of the pathogenic Th2 profile. Joint action of Treg and Th17 cells in antimicrobial immunity was suggested by models of infection with *C. albicans*, in which Treg promoted differentiation of naïve CD4^+^ T cells into Th17 cells independently of the TGF-β-mediated tolerogenic effect (250). Cereda and colleagues found that cocultured CD4^+^ T cells and autologous DCs treated with *Saccharomyces cerevisiae* (yeast) reduced the Treg rate and favored the development of Th1 effector immune response (251). To summarize, the available data point to controversies as to the role of Treg in different fungal infections.

### Parasitic Diseases

As immunoregulatory agents, Tregs play a relevant role in the prevention of adverse effects resulting from excessive immune stimulation, which might eventually be fatal (252).

In Chagas disease, a high frequency of FOXP3^+^ Treg was directly correlated with moderate inflammation, thus preventing the development of megacolon (253); a similar association was also found among patients with heart disease (254). While specific suppressor mechanisms are a continuing focus of research, the available data indicate that such cells modulate the cytokine microenvironment, IL-10 and IFN-γ in particular, and/or induce lysis of effector cells through a granzyme-dependent pathway in patients with the indeterminate form of Chagas disease (255). In C57BL/6 mice infected with *Trypanosoma cruzi*, production of endogenous glucocorticoids sustains Treg homeostasis as well as the Treg/effector T cell balance. In addition, treatment combining IL-2 and synthetic steroids induces Treg proliferation and a Th1 imbalance (256). However, discrepant data suggest that Tregs play a limited role in the pathophysiology of experimental infection with *T. cruzi*, as a slight increase in resistance against the parasite was detected after Treg inactivation in the acute stage of disease (257).

Before treatment for tegumentary leishmaniasis, the expression of Treg markers (e.g., *FOXP3* and *CTLA-4*) and IL-10 levels are elevated in tissue lesions. However, it remains to be established whether Treg accumulation is directly induced by infection or rather represents a local homeostatic mechanism to control excessive inflammation (258). In canine leishmaniasis, Tregs impair the immune response to the point of exacerbating parasite growth. At the same time, Treg recruitment prevents the development of immune-mediated disorders due to the “fine tuning” of regulatory mechanisms critical for the prevention of immune system diseases (259, 260).

An adequate immune response contributes to the control of severe malaria. Increased circulation of Treg in the peripheral blood of patients infected with *Plasmodium vivax* [e.g., through upregulation of S100A8 protein (261)] was associated with parasite load and had a suppressive effect on specific T lymphocytes (262). However, modulation of the effector response hinders the control of parasitemia by enabling uncontrolled parasite growth (263). In other Apicomplexa, such as *Toxoplasma gondii*, the basal number of Treg during acute infection does not suffice to prevent CD4^+^ T cell activation and increased production of pro-inflammatory cytokines. While Treg transfer possibly contributed to increased survival of infected mice, the number of brain cysts was increased, which points to a dual role for Treg in *T. gondii* infection and the need to maintain a delicate immune balance (264).

The persistence of helminths is also based on the breadth of Treg as a mechanism of resistance against the host immune system (265) (Figure 5). This hypothesis is based on findings showing that the Treg population expands during infection and exposure to antigens excreted or secreted by parasites, leading to changes mainly in the Th2 cell balance (266). Costimulatory molecules, such as inducible T cell costimulator (CD278), participate in the activation of FOXP3^+^ Treg and downregulation of pro-Th2 cytokines at both the local and systemic levels (lymph nodes and spleen) in some parasitic infections (267). In a model of *Trichuris muris* infection, early Treg depletion post-infection was beneficial for hosts; however, the worm burden was enhanced when Tregs were depleted later once infection was established (268).

In schistosomiasis, Tregs regulate the Th2 profile in mesenteric lymph nodes, granulomas, and fibrotic colon responses and reduce eosinophil recruitment (269). However, this might be a parasite stage-specific response, as *Schistosoma mansoni* larvae do not activate or expand FOXP3^+^ Treg during their early migratory phase (270). Imbalances in the cytokine response favorable to infection with *Schistosoma haematobium* due to increased frequency of CD4^+^CD25^hi^FOXP3^+^ Treg, among other causes, might be modified by treatment with praziquantel (271).

Infective filarial larvae (L3) recruit tTreg and induce their premature proliferation at the site of infection, rapidly biasing CD4^+^ T cell responses toward a regulatory phenotype approximately 7 days postinfection. Treatment with anti-CD25 antibodies reduced the fertility rate of parasites and the incidence of infection in experimental animals. CTLA-4, a molecule with the potential to maintain effector cell hyporesponsiveness, is also an attractive therapeutic target for reversion of protective immunity (272, 273).

Infection with *Trichinella spiralis* exhibited anti-inflammatory effects in a model of dextran sulfate sodium-induced colitis through activation of local Treg producing IL-10 and TGF-β (274). IL-10 creates an immunoregulatory microenvironment that favors the development of *Taenia solium* cysticerci and their permanence in the central nervous system. This scenario results from the mutual interaction between Treg and DCs, which induces a tolerogenic phenotype that in turn induces Treg to develop into the Tr1 phenotype (275). This feedback also occurs in *Echinococcus granulosus* infection, where the parasite inhibits DC maturation, directing them toward a tolerogenic phenotype; this may drive the differentiation of CD4^+^ T cells into Treg and suppress cytokine production in a generalized manner (276).

## FOXP3 Mutations and Their Clinical Impacts

### Scurfy

Phenotype sf corresponds to a strain of mice with a recessive mutation on the X chromosome characterized by a two base pair insertion in exon 8 of the *FOXP3* gene, resulting in the loss of the forkhead domain and nuclear localization signal; the encoded protein is truncated and non-functional (89, 277).

Hemizygous males (X*^sf^*/Y) exhibit fatal autoimmune disorders characterized by lymphoproliferative disease, with progressive infiltration of the lymph nodes, spleen, liver, and skin, causing spleen, liver, and lymph node enlargement, exfoliative dermatitis, leukocytosis, hyperglobulinemia, and severe anemia; precocious death usually occurs by the third to fourth week of life (278–281).

This mutation in the *FOXP3* gene causes deficiency of functional Treg, with consequent impact on systemic immune tolerance (282) and resulting in the infiltration of several organs with activated immune cells (283). The sf phenotype is mediated by hyperresponsive helper T cells *via* excessive production of a wide range of cytokines (IL-2, IL-4, IL-5, IL-6, IL-10, TNF-α, among others) in a generalized manner, which are associated with the typical pathological abnormalities of this phenotype (284, 285).

Expression of mutant *FOXP3* (sf) in non-hematopoietic cells in the thymus contributes to impaired thymopoiesis, which mainly affects thymocyte stages DN2 and DN4, and to a lesser degree, double-positive immature thymocytes. This, in addition to the remarkable overexpression of the *ErbB2* gene in the thymic stroma, suggests a critical role for this gene in thymic atrophy (286).

B lymphocytes and the corresponding autoantibodies worsen the autoimmune effects of hyperglobulinemia and contribute to the early death of mice due to loss of humoral immune tolerance (287). The presence of T_FR_, which were suggested to behave as direct modulators of germinal center B cells (288), controls the activation of autoreactive cells indirectly by limiting the number of follicular T cells in the center, thus limiting the activation of autoantibodies in this microenvironment, although it does not rule out the possibility that T_FR_ exert a direct control over the activation of B cells (287, 289).

While neonatal thymectomy increases the survival of sf mice and attenuates their immune and clinical state, it cannot prevent the appearance of pathological manifestations derived from the probable generation of fetal T lymphocytes that migrate to other parts of the body, thus supporting the development of the sf phenotype (280). Partial bone marrow transplant and one single infusion of T-enriched splenocytes might rescue the autoimmune sf mouse, which indicates that sf effector T cells are susceptible to dominant regulation by a small fraction of cells *in vivo* (290).

### Immunodeficiency, Polyendocrinopathy, and Enteropathy X-linked

A phenotype analogous to murine sf in humans is exhibited by patients with the IPEX syndrome. In this condition, Treg dysfunction causes several fatal autoimmune disorders affecting the intestines, skin, endocrine organs, and blood. Clinically, patients present neonatal insulin-dependent diabetes mellitus, chronic refractory diarrhea, severe food allergies, thrombocytopenia, hemolytic anemia, chronic dermatitis with bullous pemphigoid (in the rarest cases), hyperthyroidism, and lymph node and spleen enlargement (283, 291–297).

A wide variety of autoantibodies are detected in a large portion of IPEX patients; some of them might directly affect target organs, while others might be unrelated to the pathophysiology of disease (293, 298). Specific antibodies against autoimmune enteropathy-related antigen (AIE-75) and protein villin—both antigens expressed in the intestinal microvilli and renal proximal tubule—are frequently detected in the serum of IPEX patients and are related to activation of the humoral response and catastrophic local tissue damage (299–301). Specific antibodies against pancreatic islet cells, insulin, or anti-glutamate decarboxylase are indicative of neonatal type 1 diabetes; anti-thyroglobulin and anti-microsomal antibodies are related to autoimmune thyroiditis (302). Anti-mitochondrial antibodies are typically associated with primary liver cirrhosis in adults (298).

The first study that linked the IPEX syndrome with genetic variations in *FOXP3* was devised by Bennett et al., in which the authors identified distinct mutant profiles in the gene in two affected families (303). Currently, about 63 mutations in the *FOXP3* gene related to the etiology of IPEX have already been reported (303), mainly affecting the forkhead domain, the LeuZip domain, and the protein N-terminal portion (21). Mutations in the polyadenylation site lead to unstable *FOXP3* expression, which is associated with early worsening of disease (304). Similar complications occur in carriers of missense mutations due to abnormalities in the expression of the mutant protein (305).

Treatment options for IPEX are limited and are based on individual cases. General measures include blood transfusion, insulin replacement therapy, antibiotic prophylaxis, and immunosuppressive agents, which mostly only afford transient relief (296, 305). However, a recent study by Chen and colleagues found that rituximab monotherapy or combined with tacrolimus was effective in inducing suppression of Th2 cells, which mediate gastrointestinal and kidney lesions in the IPEX (306). Functional cure is achieved through hematopoietic stem cell transplantation, especially when performed in the first years of life (307); both myeloablative and non-myeloablative conditioning regimens are used to avoid complications associated with transplantation (302).

### Genetic Polymorphisms

In addition to the aforementioned classic mutations, the immunological relevance of polymorphisms in various regions of the *FOXP3* gene is being investigated.

As the promoter region is involved in initiating transcription as well as in the interaction with *cis*-acting elements that regulate gene expression, it was suggested that this region might harbor functionally relevant polymorphisms. Such polymorphisms might eventually reflect on the *FOXP3* expression level and consequently on Treg activation, as in the case of autoimmune diseases (308). Five single-nucleotide polymorphisms (SNP) have been described in the *FOXP3* promoter region: −924A/G (rs2232365), −1383C/T (rs2232364), −2383C/T (rs3761549), −3279C/A (rs3761548), and −3499A/G (rs3761547) (309, 310).

Binding site analysis of the −924A/G (rs2232365) SNP showed that it interacts with the transcription factor GATA-3, which plays an essential role in Th2 responses; the presence of the mutant G allele was suggested to interfere with this interaction. The −924A/G (rs2232365) SNP was significantly associated with the occurrence of spontaneous abortion in a Chinese Han population (311), psoriasis (312), and chronic rhinosinusitis (313); this association was not confirmed for Crohn’s disease (314) or Graves’ disease (26). Mutant haplotype carriers of rs2232365, rs3761548, rs5902434, and rs2294021 SNPs exhibited 2.5 times higher risks of recurrent idiopathic abortion (315). Combined analysis of variants rs2232365 and rs3761548 showed that carriers of mutant alleles in specific subgroups of the Han people exhibited higher risk of vitiligo (316).

Functional differences in the −2383C/T (rs3761549) SNP were first suggested in the study of Inoue and colleagues, who found low *FOXP3* expression levels and decreased Treg suppressor function in genotype CC carriers. This reduction in regulatory function might increase the activity of autoreactive T cells accompanied by severe destruction of the thyroid tissue in patients with Hashimoto’s disease (317). The −2383C/T (rs3761549) SNP, in association with the FCRL3 gene (318), was also related to the occurrence of endometriosis, independently of the stage of disease (319), with a predisposition to Graves’ disease (320) and food allergy development (321); the T allele was rated as a risk factor for SLE in a Chinese population (322). The −3499A/G (rs3761547) SNP was also studied by Inoue and colleagues in relation to the prognosis of Hashimoto’s and Graves’ disease, but no significant differences in the genotypic or allelic frequencies of this polymorphism were detected (317), similar to the case of juvenile idiopathic arthritis (323).

Shen and colleagues suggested that carriers of the AA genotype of SNP −3279C/A (rs3761548) have abrogated binding to the E47 and c-Myb transcription factors, which results in defective transcription of FOXP3 (324). A considerable number of published studies have associated this polymorphism with diseases and other conditions that reflect Treg deficiency, such as allergic rhinitis (325), susceptibility to vitiligo (326), psoriasis (327), some autoimmune diseases (328), non-small-cell lung cancer (329), and breast cancer (330). This association has not been confirmed for other disorders, including endometriosis-associated infertility (319) and systemic sclerosis (331).

Microsatellite polymorphisms (GT_n_) in the *FOXP3* promoter region were also investigated. Ban and colleagues found an association between this type of polymorphism and thyroid autoimmune diseases in a European, but not Japanese, cohort (332). Another study found a positive relationship between this polymorphism and survival in kidney transplant recipients (333). However, the presence of GT_n_ was considered irrelevant for susceptibility to SLE, rheumatoid arthritis, inflammatory bowel disease, and celiac disease (334).

Some SNPs have been described in the *FOXP3* intronic region, such as −20A/G (rs2232368), +87G/T (rs2232366), +459C/T (rs2280883), +459C/T (IVS9), among others (310). Analysis of the rs2232368 and rs2280883 polymorphisms revealed a possible association with idiopathic infertility; a similar relationship was not found for the rs2232366 polymorphism (319). The rs2280883 variant was associated with susceptibility to systemic sclerosis in Italian patients (331), and its genotypic frequency exhibited significant differences in patients with primary biliary cirrhosis (335). The mutant TT genotype was found to be more frequent among patients with hepatitis B-related hepatocellular carcinoma (336).

The +459C/T (IVS9) SNP was associated with a risk of myasthenia gravis in a Han Chinese population, with wild allele C being a protective factor against this condition (337). In turn, genotype CC was associated with a risk of severe psoriasis also in a Han Chinese population (327), and this effect was age-dependent; associations of this polymorphism with lung cancer were found among aged patients (338).

Four further intronic polymorphisms were studied by Bottema and colleagues: variants rs5906761 (upstream of exon 1) and rs2294021, rs2294019, and rs6609857 (located at the 3′-end). These authors found an association between the investigated SNPs and allergic sensitization to egg among girls aged 1–2 years old. In turn, polymorphisms rs5906761 and rs2294021 were associated with remission of sensitization to food allergens in boys (321).

Few studies have investigated the association between exonic variants and modulation of FOXP3 expression. Kim and colleagues analyzed the coding regions of exons 2–12 in acute leukemia patients but failed to detect any mutations. According to these authors, mutations in FOXP3 occur exclusively in some types of tumors, such as breast and prostate cancer (339). The study of Bafunno and colleagues did not find any association between polymorphisms in exon 12 and the formation of neutralizing antibodies in hemophilia A (340).

## Conclusion

Mechanisms that regulate the immune response are essential in maintaining the homeostatic balance between the immune activation necessary to give combat to a specific manifestation and the control of possible damages generated by the exacerbated expression of this response. The present review brings together a number of relevant studies on the role that Treg and FOXP3 play as modulating mechanisms in the immunological response. It is argued that these factors actively participate in the immunoregulation of both pathological conditions and in more specific immunotolerance contexts. Thus, the discrepancies related to the functional benefits of these factors are dependent on the context discussed, since immunoregulation may favor immunotolerance and decrease the tissue aggression generated by the immune response. It is relevant to mention that it provides mechanisms of immune escape to infectious agents or microenvironments permissible to the carcinogenic activity. Efforts are being made to understand new therapeutic platforms involving the potential suppressor of Treg in stimulus models and by direct transfer of these cells, in addition to studies that investigate whether genetic variations in the *FOXP3* gene may be risk factors for the involvement of other pathologies.

## Author Contributions

LP, SG, RI, and AV contributed equally to the design and writing of the present review.

## Conflict of Interest Statement

The authors declare that the research was conducted in the absence of any commercial or financial relationships that could be construed as a potential conflict of interest.
